# Working Well in a Dynamic Nursing Career: A Critical Incident Technique Study of Nurses’ Actions for a Sustainable Working Life

**DOI:** 10.1155/jonm/1162387

**Published:** 2026-06-16

**Authors:** Johanna Gustafsson, Bengt Fridlund, Elisabeth Ericsson

**Affiliations:** ^1^ School of Health Sciences and School of Behavioral, Social and Legal Sciences, Örebro University, Örebro, Sweden, oru.se; ^2^ Centre for Interprofessional Collaboration Within Emergency Care (CICE), Linnaeus University, Växjö, Sweden, lnu.se; ^3^ School of Health Sciences, Örebro University, Örebro, Sweden, oru.se

## Abstract

**Background:**

A sustainable working life for nurses involves creating conditions that allow productivity and well‐being to be maintained throughout a nursing career. To address nurses’ health and well‐being, as well as the ongoing shortage of nurses, it is essential to understand the key elements that contribute to a sustainable working life for nurses.

**Objective:**

The aim was to explore and describe nurses’ actions in situations that are important to sustainability in their working lives, as well as why these actions matter and how they contribute to sustainable working life across the career span.

**Design:**

An exploratory descriptive design using a qualitative research approach based on Flanagan’s critical incident technique (CIT).

**Setting and Participants:**

Forty‐seven nurses (general and specialist) from diverse fields of care within three healthcare regions in Sweden were interviewed between April and October 2024.

**Methods:**

A two‐step sampling approach was used. Data were analyzed inductively, in line with the guidelines for CIT.

**Results:**

Nurses employed various individual‐, organizational‐, and team‐oriented strategies to maintain a sustainable working life. Organizational support played a vital role. Supportive leadership adopting a “person‐first” approach and a positive organizational culture facilitated work–life balance, professional development, and effective teamwork. Negative organizational cultures, such as excessive workloads and ethical conflicts, reduced nurses’ well‐being and willingness to remain with the organization.

**Conclusions and Implications for Nursing Management:**

Sustainability in nursing emerged as a dynamic process shaped by the interaction between nurses’ adaptive strategies and organizational conditions, particularly supportive leadership and a positive organizational culture. This underscores the importance of integrated, long‐term management approaches that balance individual needs, professional development, and supportive work environments to sustain nurses’ engagement across the career span.

## 1. Introduction

Sustainable working life has emerged as a key concept in addressing long‐term workforce challenges across healthcare systems globally. Unlike traditional retention perspectives that focus on whether employees remain in an organization over a defined period, sustainable working life adopts a life‐course approach, which aims to ensure that “working and living conditions are such that they support people in engaging and remaining in work throughout an extended working life” ([1], page 2). This perspective necessitates ongoing alignment between organizational conditions and individuals’ evolving capacities, resources, and life circumstances. While the conceptual framework is still being clarified [2], it highlights the dynamic interactions between structural work conditions and individual trajectories over time.

The relevance of sustainable working life is particularly evident in nursing. As reported, healthcare systems face significant challenges, such as nursing shortages. The World Health Organization predicts a global shortage of 4.6 million nurses by 2030 unless immediate action is taken [3], and there is declining interest in pursuing nursing careers among young people [4]. Across Europe and other high‐income countries, nurse shortages and high turnover rates continue to challenge healthcare systems. Sweden represents one example, with turnover reaching 13.9% in 2023 [5] and shortages affecting numerous specialties [6, 7]. These figures demonstrate that short‐term retention strategies are insufficient. Ensuring sustainability in nurses’ working lives requires a long‐term, systemic perspective that supports nurses’ health, productivity, and commitment to nursing over time. Within nursing, sustainable working life can therefore be conceptualized as an integrative framework that connects sustainable organizational conditions—such as work organization, resource adequacy, a relational climate, and leadership practices—with nurses’ health and well‐being as interdependent mechanisms promoting long‐term participation in the nursing profession.

Linked to sustainability in working life is the crucial issue of the well‐being and retention of nurses. Basso et al. [8] underscore the relationship between nurses’ well‐being and their intentions to leave the profession. Intention to leave reflects self‐reported attitudes toward remaining in nursing and is considered a strong predictor of actual turnover, with implications for future retention. Their international review [8] highlights the multidimensional nature of such intentions. Low job satisfaction, high workload, inadequate workplace social relationships, and poor leadership are central factors behind the intention to leave, as are insufficient professional and economic recognition. Moreover, stress, burnout, and deteriorating physical, mental, and social health further increase this risk.

Furthermore, research has examined the reasons for nurse turnover, with evidence suggesting that intentions to leave a particular organization are linked to organizational conditions, whereas decisions to exit the nursing profession are primarily associated with individual‐level factors [7]. The significance of organizational conditions is further underscored by a Swedish study identifying five categories of reasons nurses gave for leaving the profession: lack of compensation and perceived unfairness, the psychosocial work environment (mental and emotional strain), limited career development opportunities, dissatisfaction with nonclinical workload, and insufficient support and safety [10]. This was especially prominent for younger nurses and nurses in non‐primary care settings.

Intention to stay constitutes a central construct within sustainability research, as it is positively associated with future retention and nurses’ willingness to remain in the profession and/or at the workplace. The literature consistently demonstrates that organizational‐level factors play a decisive role in strengthening nurses’ intentions to remain in nursing. High‐quality work environment, support systems, and organizational climate serve as protective factors that enhance nurses’ willingness to stay in their jobs [11]. The practice environment [7] is also important for nurse retention, as is the relational climate, including supervisor and organizational support [12]. Leadership style—conceptualized as managerial behaviors and decision‐making patterns that shape nurses’ formal working conditions—represents one organizational mechanism influencing retention [13]; another is leader–member exchange (LMX), which captures the relational quality emerging in interactions between leaders and individual nurses [14]. Considered together, these structural and relational aspects of leadership help explain how organizational environments can foster or undermine nurses’ long‐term engagement in the profession. Furthermore, other organizational factors play a protective role. Commitment, for example, which is characterized by a sense of belonging, job satisfaction, loyalty, and responsibility toward professional duties [15], has been found to contribute to sustained engagement in the nursing workforce [16].

Retention is further conditioned by situational and contextual factors, reinforcing its relevance within a sustainability framework. Specific care settings [17] and extraordinary circumstances such as the COVID‐19 pandemic [18], illustrate how environments characterized by sustained emotional, cognitive, and physical demands can either undermine or strengthen nurses’ capacity to remain in the profession, depending on the availability of structural and relational support. From a sustainable working life perspective, this indicates that retention is not simply an individual decision, but the outcome of how organizational conditions interact with nurses’ health and resources over time. Although findings vary across contexts, the accumulated evidence underscores that enhancing organizational quality and adaptive capacity is fundamental to enabling sustainable professional engagement across the career span.

Health and well‐being are central to the concept of sustainability in the nursing profession. Although there is no single conceptual model or theoretical framework dedicated to nurses’ organizational well‐being [19], the evidence clearly demonstrates that health‐related factors decisively influence nurses’ working life. For newly qualified nurses, negative health outcomes such as emotional exhaustion and stress tend to decrease as job satisfaction increases over time [20]. Furthermore, positive health resources such as resilience, optimism, and hope tend to improve with professional experience. These findings suggest that sustainability is not merely about preventing early exit, but also fostering trajectories in which health resources are gradually built and stabilized.

Despite this insight, the research base remains fragmented, as much of the existing empirical research on nurses’ health and well‐being tends to focus on specific care settings or specialties (e.g., critical care; [21]), particular groups (e.g., new nurses; [20, 22]), or specific situations, such as the COVID‐19 pandemic [23, 24]. Broader profession‐wide analyses of nurses’ health and well‐being across the profession are comparatively scarce and are often confined to intervention reviews (e.g., [25, 26]). In addition, as the latter review indicates, most interventions focus on the individual, with less emphasis being placed on organizational strategies that modify everyday work practices and protocols to enhance employee well‐being. This imbalance highlights a critical gap in relation to sustainable working life. If sustainability concerns the ability to remain healthy and professionally engaged over time, it cannot primarily rely on individual coping strategies that address immediate mental and emotional strain. Such approaches overlook how structural conditions accumulate and exert effects across the career span.

Although determinants of nurse retention and well‐being are well documented, the literature remains fragmented and is largely characterized by short‐term and cross‐sectional perspectives. Studies often examine isolated factors—such as leadership, job control, or mental health—without integrating them into an overarching sustainability framework. This limits theoretical development and risks reducing retention to a question of short‐term stabilization, rather than an attempt to understand sustainability across the career span. Furthermore, limited attention has been given to how nurses actively navigate and respond to situations that shape their long‐term professional engagement. By examining nurses’ actions in sustainability‐relevant situations and identifying the organizational, relational, and individual conditions that enable professional continuity, this study contributes to a more integrative and dynamic understanding of sustainable working life. Such knowledge is needed to move beyond fragmentation and short‐term initiatives toward systemic approaches that support nurses throughout their professional life course. To address this gap, the present study aims to explore and describe nurses’ actions in situations that are important to sustainability in their working lives, as well as why these actions matter and how they contribute to sustainable working life across the career span. By adopting such a sustainability perspective, the study seeks to advance a more integrated and dynamic understanding of sustainable working life in nursing.

## 2. Material and Methods

This study employed an exploratory and descriptive approach, using Flanagan’s [27] critical incident technique (CIT) in line with the framework proposed by Fridlund et al. [28, 29]. Evolving from pragmatic realism toward a constructivist, human‐centered epistemology, CIT assumes that knowledge about practice emerges through systematic analysis of participants’ narrated actions and their consequences [29], making it particularly suitable for examining how nurses’ situated actions shape sustainable working life over time. Given that sustainable working life concerns how professional engagement is maintained or undermined over time, it is essential to identify not only conditions but also the specific actions nurses take in response to sustainability‐relevant situations. By focusing on retrospective accounts of significant situations, CIT allows participants to articulate what they did, why they acted as they did, and what consequences followed, which facilitates an understanding of the behaviors and capabilities of nurses in these situations. CIT’s action‐oriented approach aligns with the study’s aim to understand how nurses actively navigate situations that influence sustainability in working life. The method has been used within the same field, e.g., to analyze nurses’ intention to leave and final decision to quit their job at a hospital unit [30].

### 2.1. Setting

Data were collected through interviews with nurses from three different healthcare regions in Sweden, including university hospitals, municipal care facilities, and primary care centers offering both specialized and general healthcare services. The Swedish healthcare system is publicly funded and primarily tax‐based, with healthcare services organized and delivered by 21 self‐governing regions. Care delivery models vary across settings, and no single national nursing care model is mandated. Staffing levels, including nurse‐to‐patient ratios, are not regulated by national legislation, but are determined at regional and unit levels. Consequently, ratios vary depending on care context and local resource allocation.

Nurses’ working conditions are regulated through collective agreements negotiated by unions. Full‐time employment typically corresponds to approximately 38–40 h per week, depending on shift schedules and agreements related to night and weekend work. The average monthly salary for registered nurses varies by region and experience level. In 2024, the mean salary per month was SEK 40,031 for a general nurse and SEK 44,773 for a specialist nurse [31]. Nurses are covered by occupational pension schemes in addition to the national public pension system, with a flexible retirement age currently beginning in their mid‐60s.

### 2.2. Sampling

Sampling was conducted in two stages. Initially, a convenience sampling approach was used to access a pool of 1020 nurses who had participated in the project *“Healthy and Sustainable Working Life for Nurses in Swedish Healthcare.”* From this pool, participants were purposefully selected to ensure variation in characteristics such as care field and years of experience. Nurses were eligible for inclusion if they were currently employed in healthcare, either public or private, and were able to participate in an interview conducted in Swedish. Invitations with detailed information about the study were sent by email to nurses with different characteristics (age, gender, level of education, years of experience, fields of care, and healthcare settings). In line with CIT, the unit of analysis was situations rather than individual participants [28]. Sampling was therefore guided by the aim of generating a sufficiently large and varied pool of sustainability‐relevant situations. Based on the methodological recommendation that approximately 100 incidents are required to achieve stable category development [28], it was anticipated that each participating nurse would describe two to three important situations. Consequently, a large initial pool of nurses was invited to ensure that an adequate number of sufficiently diverse critical situations could be obtained for robust analysis. The invitations resulted in 59 responses and 47 actual participants. The main reasons for nonparticipation were time limitations and lack of response to follow‐up communications regarding interview scheduling.

Before the interviews, participants received comprehensive information about the objectives of the study and instructions on how to describe an important situation. As suggested by Fridlund et al. [28, 29], they were prompted to include details such as the beginning and end of the event, as well as its tangible outcome, whether positive or negative. An example from a different context, such as family life, was provided to clarify the type of information being sought. This preparatory step aimed to ensure accurate and detailed recollections during the interviews.

To ensure transparency and methodological rigor, the authors adhered to the Consolidated Criteria for Reporting Qualitative Research (COREQ) guidelines [32] and comprehensively documented the study design, team composition, analytical methods, and findings.

### 2.3. Participants and Data Collection

The study involved interviews with 47 nurses, each with diverse experiences from various healthcare settings (see Table 1). The participants were between 34 and 66 years old (mean 50 [SD ± 9]), and most were female (37/79%) and worked in tertiary hospitals (36/76.6%). Regarding education level, 11 (23.4%) nurses had an undergraduate degree. One (2.2%) was a radiographer. 35 (74.4%) nurses had postgraduate academic degrees, among which 26 had a specialist nurse exam, and six nurses had specialist education in various medical specializations. Three participants were midwives. Most of the nurses had a master’s degree (*n* = 27/57.4%). The average clinical work experience as a nurse ranged from one to 42 years. Regarding work hours, most of the nurses worked full‐time (38 [81%]).

**TABLE 1:** Nurses’ characteristics (*N* = 47).

	Frequency	Percentage
Gender		
Female	37	79
Male	10	21
Age mean 50.1 (SD ± 9.0) years old		
30–36	4	8.5
36–45	13	27.7
46–55	15	31.9
56–60	9	19.1
61–65	5	10.6
66–70	1	2,1
Education		
Radiology nurse/radiographer	1	0.02
Undergraduate nurse with no specialist education	11	23.4
Postgraduate nurse:	35	74.5
Specialist education focus on cardiovascular (*n* = 1), nephrology (*n* = 1), neurology (*n* = 1), diabetes (*n* = 1), pulmonary care (*n* = 1), urology (*n* = 1)	6	12.8
Midwife	3	6.4
Specialist nurse in medical care (*n* = 2), pediatric care (4), psychiatric care (*n* = 5), surgical care (*n* = 1), community health (*n* = 10), geriatric (*n* = 1), occupational health nurse (*n* = 2), emergency medical care (*n* = 6), intensive care (*n* = 2), anesthesia (*n* = 1), ambulance care (*n* = 2). Eight nurses had several postgraduate diplomas in specialist nursing.	26	57
Academic exam		
Candidate	19	40.4
Master	27	57.4
PhD	1	2.1
Workplace		
Municipal health care	2	4.3
Primary hospital	9	19.1
Tertiary hospital	36	76.6
Work‐time		
Full‐time	38	80.9
Part‐time	9	19.1
Number of years as a nurse		
Mean 21.0 (SD ± 11.3), range 1–42 years		

Interviews were conducted by the first author, a behavioral scientist with long experience in qualitative research. The interviewer had no prior relationship with the participants; they were informed about the interviewer’s role as a researcher. Interviews were conducted in a private setting via a digital platform. Before the interview started, participants were informed about the confidentiality procedures and their right to withdraw at any time, and given digital informed content.

The interviews lasted between 45 and 80 min. Participants were instructed to focus on a single important situation at a time and emphasize its impact on sustainability in their working life. They were asked to provide a detailed description of the situation, including the experiences and actions that had influenced the outcome. An interview guide (Table 2), adapted by Fridlund et al. [28], was used to facilitate responses, after having been tested in pilot interviews to ensure its rigor and suitability for this setting. The interviews yielded rich data reflecting the complex and multifaceted nature of nurses’ actions and strategies in important situations affecting their work–life sustainability.

**TABLE 2:** Interview guide according to the Fridlund et al.^∗^ framework for critical incident technique interviews.

Opening statement: Please describe, concisely, an important situation for sustainability in your working life as a nurse.
Standardized questions:
Can you give a detailed description of what happened?
What did you/others do in connection with the important situation?
Where did the important situation take place?
Why was the situation important to you?
What was your attitude at the time of the situation?
What were you thinking during and after the situation?
How did you feel during and after the situation?
What did you find most challenging about the situation?
What has this situation meant for you going forward?

^∗^Fridlund B, Henricson M, Mårtensson J. Critical incident technique applied in nursing and healthcare sciences. SOJ Nurs Health Care. 2017;3 (1):1–5.

All interviews were audio‐recorded using a digital recording device to accurately capture participants’ accounts. The recordings were transcribed verbatim by a professional transcription service. During transcription, all identifying information (e.g., names of individuals, workplaces, and locations) was removed to ensure confidentiality prior to analysis.

Field notes were written during the interviews and documented immediately after each interview to capture observations and preliminary analytic reflections. These notes were used to support interpretation during the analytic process, but were not treated as primary data. Transcripts were not returned to participants for review, but during the interviews, the interviewer summarized each described situation to ensure an accurate understanding. Participants were given the opportunity to confirm, clarify, or elaborate on the summary, thereby enhancing the credibility and accuracy of the recorded situations.

### 2.4. Data Analysis

The analysis followed an inductive approach aligned with CIT and focusing on the nurses’ experiences and actions in important situations, as outlined by Fridlund [28, 29]. The objective was to identify nurses’ actions in situations important to sustainable working life and to examine the perceived consequences of these actions.

In the first step, the first author conducted multiple readings of all interview transcripts to identify descriptions of situations perceived by participants as decisive for sustainability in nursing. A critical situation was defined as a clearly delineated situation containing (a) contextual conditions, (b) an observable action undertaken by the nurse, and (c) perceived outcomes and consequences. For example, a nurse’s request for managerial support to prevent burnout during a period of excessive workload was identified as an important situation. Only situations meeting all three criteria were included in the analysis to ensure methodological rigor and analytic consistency. The co‐authors independently reviewed a subset of the transcripts, after which the identified situations were discussed collectively to reach consensus on inclusion according to the predefined criteria, thereby strengthening credibility and analytical rigor.

In the second step, actions within each situation were extracted and analytically linked to their reported sustainability‐related outcomes. Outcomes, as described by the nurse, were classified as either sustainability‐enhancing (e.g., strengthening the nurse’s intention to remain in nursing) or sustainability‐undermining (e.g., strengthening an intention to leave).

The analysis then proceeded with continual comparison across situations to identify recurring patterns of action and their associated consequences. Recurring patterns were defined as conceptually similar sustainability‐related behaviors described by multiple participants across different contexts. These patterns were grouped into subcategories (e.g., “Changing jobs”) based on shared functional characteristics. Subcategories were subsequently clustered into broader categories (e.g., “Self‐management of the work situation”) reflecting higher level conceptual similarities. This process involved iterative comparison between empirical data and emerging categories. Disagreements regarding categorization were resolved by systematically re‐examining the original transcripts until consensus was reached. Finally, interpretative abstraction resulted in main themes reflecting nurses’ actions to achieve a sustainable working life across the career span. The main areas were developed by identifying higher order conceptual linkages between categories that reflect shared functional meanings in relation to sustainability (e.g., individual‐oriented strategies). Table 3 provides examples to illustrate the process of analysis.

**TABLE 3:** Example of the analytical process from situation to main area.

Situation as a narrative	Extraction	Sustainability‐related outcomes	Subcategory	Category	Main area
“I started in 24/7 care. It was quite a shock, almost like a slap in the face — ‘Oh, is this really how it’s supposed to be?’ I was actually very surprised by how difficult it was. There were also small things that I reacted to. I remember being surprised that there wasn’t even a computer available when you arrived and needed to log in. I became stressed about that — “Oh God, I need to find a workstation.” The computers were not fixed workstations but laptops, so you had to search for one in order to read about the patients and prepare. I remember thinking, “There aren’t even enough computers.” Eventually you might find one, but the situation was very stressful. I also found the systems difficult to use. IT systems are important—they need to be well designed for you to do your job properly. I remember reflecting on a small tab in the system we use at the hospital, called Cosmic. It was very small, and that was where you had to check whether a patient was incoming. For me as a nurse, that was very important information if I needed to prepare something, insert an IV line, or take samples. But it was very easy to miss. I remember wishing that we had received a different kind of introduction to the system, or that the system had been designed differently. “I stayed there for a few months and continued working, but I remember that period as very stressful and demanding, especially when working evening and day shifts.” /…/I remember that period as extremely demanding. My shift was supposed to end at 9:30 p.m., but it often ended at 10:00 or even 10:15 p.m., which meant that all the grocery stores had already closed except for the small convenience store on the corner if you needed to buy something after work. Then you would try to go to sleep without waking the children—we had a small daughter at the time—and then get up and be at work at 6:45 a.m. You absolutely did not want to be late, because then the night staff would be upset. I remember that period as very exhausting, and it was not sustainable for long. I started in October and left in May, so I stayed a little more than 6 months. I also remember one particular event. I had had a very difficult day at work, with no lunch break, and afterward I went to my father’s workplace. He made me some sandwiches and a cup of tea, and I just broke down. He said to me, “Work shouldn’t be like this. You have to leave that job, otherwise you will burn out, and it can take a long time to recover from that.” That conversation helped me gain some perspective, and after that event I actually resigned. It was not difficult to find a new job—there are many nursing jobs available, although you may not want all of them. I moved to a daytime position in elder care. I remember thinking, “This may not be as interesting, but at least it offers better pay and better working hours.” When I started working there and regained regular working hours—the kind of rhythm you have since school, getting up on Monday morning and going to work—it almost felt like getting my life back.”	The work was stressful due to insufficient organizational resources, particularly a lack of supportive systems, and difficulties in completing tasks within scheduled working hours. This led to challenges in maintaining a work–life balance. After experiencing an emotional breakdown in a safe environment, the nurse decided to change jobs and moved to a daytime position that was less stimulating but provided a more stable daily rhythm.	Job change as a strategy for remaining in the nursing profession	Changing jobs	Self‐management of the work situation	Individual‐oriented strategies

“The reason I left inpatient care was that… well, I worked there from 2008 to 2010, and in 2010 something shifted. It never really stops abruptly, but at some point it becomes decisive. When you rotate between four care teams during the same week, meeting about 12 patients in each team, and each patient has at least 10 different things you need to keep track of—breathing and circulation, underlying conditions, medications, tests, how they are feeling, and so on. It is one thing to have 12 patients, but when you move between four teams that means around 50 patients in a week, and roughly five hundred parameters to keep track of. It becomes quite chaotic, and at some point you reach your limit.” At the same time, you switch between evening, day, and night shifts within the same week, and no one ever says, “We should probably not ask you to take an extra shift.” It was almost taken for granted. You might work two evening shifts or two night shifts in a row, finish on Tuesday morning, then work day shifts Wednesday, Thursday, and Friday, and still be asked, “Could you take the weekend? We’re short staffed.” No one looked back to see what you had already done. Eventually I had had enough. You are not an unlimited resource, and at some point you need recovery. Unfortunately, sometimes you have to reach that point to understand where your limits are. I finally said, “I can’t do this anymore. I’m going home.” After that there were meetings with HR and other things… “Later I moved to this job. There I was mostly sitting at a computer, handling one call at a time in a quiet and calm environment. Only then did I realize how heavy the workload had been before. When you are in that kind of work situation you do not really see it—you just try to solve things in the moment and keep going. You have to step out of that environment to understand how demanding it actually is.”	Work in inpatient care was complex, involving multiple simultaneous processes and extensive responsibility for patient care. Management frequently requested additional shifts without reflecting on nurses’ existing workload, resulting in insufficient opportunities for recovery. The organization of the work eventually led the nurse to feel unable to continue, prompting a move to a workplace where tasks could be handled one at a time and where recovery was possible.	Job change as a strategy for remaining in the nursing profession	Changing jobs	Self‐management of the work situation	Individual‐oriented strategies

“I had been working alone for quite a long time. A colleague became sick‐listed due to exhaustion, and I started to think that I might also be at risk. I don’t know if I actually was, but I found myself wondering, ‘How long will I be able to keep going, and what is it that helps me cope?’ Those thoughts were probably one of the reasons why I decided to leave. I felt that this situation was not sustainable and that I had to do something else.” I had a lot of responsibility for our company clients and their visits, and I realized that I could not continue working like that for the remaining years of my career. That made me start looking for other jobs and be open to new opportunities. At the time I was about to turn 60, and I knew that it becomes more difficult to change jobs the older you get. I wanted a position with daytime hours, no weekends, and where I could be part of a different work environment, because I felt that the isolation in my previous job was not good for me. “Then a position at a child health center was advertised, and I thought, “Yes, I can apply for this”. And I got the job (laughs).”	Working alone and experiencing an increasing workload led the nurse to perceive a risk of exhaustion and to feel that continuing under such conditions was not sustainable for the remaining years of working life. As a result, the nurse changed jobs and moved to a daytime position with colleagues.	Job change as a strategy for remaining in the nursing profession	Changing jobs	Self‐management of the work situation	Individual‐oriented strategies

During each of these steps, the analytical process involved reflective discussions with all the authors to enhance credibility. These discussions included consensus‐reaching on the inclusion of situations according to the predefined criteria, on the recurring patterns, as well as on the categorization and main themes.

### 2.5. Ethical Considerations

The study was approved by the Swedish Ethical Review Authority (DNR 2023‐02197‐01, 2023‐02962‐02) and the research adhered to the principles outlined in the Helsinki Declaration [33] and the General Data Protection Regulation (GDPR). Participants provided informed consent after receiving thorough information about the aim of the study, ethical considerations, the voluntary nature of their participation, and their right to withdraw at any time. Confidentiality was maintained throughout the data analysis and reporting, and findings were summarized at the group level and supported by quotations to illustrate key points.

### 2.6. Findings

The findings demonstrate how nurses employ individual‐, professionalism‐, and team‐oriented strategies to manage a sustainable working life as a nurse. In total, 196 actions were described, most of which were related to individual‐oriented strategies, followed by professionalism‐oriented strategies and team‐oriented strategies. Table 4 provides an overview of the number of actions in the main areas, categories, and subcategories, and Table 5 provides an overview of findings, with citations.

**TABLE 4:** Citations of nurses’ actions.

Citation	Subcategory	Category	Main area
… I get to spend time with my children and take them to soccer and floorball practice. After school, I can go to the swimming pool, for example, because I have the day off after working the weekend before. So, more time with the kids is the positive outcome.	Adjusting work hours for personal reasons	Self‐managing the work situation	Individual‐oriented strategies
And I worked hard there and I was so close to hitting the wall, but I was pregnant and I ended up going home and having a baby and taking parental leave, and then I got some perspective on the whole thing and quit my job and didn’t go back.	Changing jobs		
So then I realized that if I’m going to last in this field where, when it comes down to it, no one really cares about the staff, then I’ll have to find another way to deal with it. And so I did that by getting a private therapist so that I could solve all the problems.	Receiving support from counseling professionals		
And that’s when I signaled to her that this isn’t working for me, that I have so little to do/…/She took it seriously, like, “Is it that you feel so… tired of just coming here and sitting around, that your body isn’t feeling well?” “Yes, that’s how it is,” so I had originally intended to take one day off a week using vacation time, but then she said, “You shouldn’t have to do that.” There’s another place where they really needed more help, and one day a week would make a big difference for them.	Communicating about personal matters with immediate superior	Shared responsibility for the work situation	
But when I decided to leave, I contacted my boss here/…/to get references, and then they understood that I was on the market again/…/They asked if I was interested in a position if they adapted it to my wishes at the time. And then they did that, and I actually accepted it.	Customizing the work conditions		

And then I just felt, “This is completely impossible – I can’t leave my two seriously ill patients and my fairly recently graduated nursing colleague/…/I can’t take responsibility for this.” “Yes, but we don’t have any other solution, so… we’ll just have to keep our fingers crossed that there won’t be any alarms.” And so I had to wear that pager all day, and it was like someone had punctured my nursing soul. After that, I didn’t know up from down, and I didn’t return to work after that.	Prioritizing the patient’s care needs	Adhering to a patient‐first approach	Professionalism‐oriented strategies
… I try to get to know both the relatives and the person I’m caring for to prevent situations like this that are so stressful and make everyone feel bad/…/if you’ve managed to establish some kind of relationship and contact and you follow each other and have that communication of information/…/then usually, anything can happen without it turning out like it did at the beginning of this example I described, when they just stand there screaming.	Tailoring care to patient’s needs and preferences		
…was offered the opportunity, after specialization, to take a step up the career ladder/…/it was about her creating a job role for me that was actually person‐centered, based on my qualities and also areas where I needed to improve (laughs)	Taking career growth opportunities	Continuing professional development in nursing	
…writing down a few different ways and tackling this in a low‐affect way, saying, “Yes, my role here is to make medical assessments based on level of urgency, so unfortunately, with this level of urgency, you will have to come to a health center the next day. So, unfortunately, I can’t give you anything else.” And then I had three or four different, really long possible ways to comment on this/…/And then I kept that paper with me until I felt calmer about it.	Creating a set of procedures for successful nursing practice		

Then when we were done there, we gathered… well, you’re supposed to have a debriefing, but I didn’t think that felt right. I didn’t get anything out of it personally, because we mostly talked about what hadn’t gone well. That’s completely uninteresting in that situation. Unstructured and uninteresting. And after that I went home, where my whole family was sitting there with boyfriends and so on, wanting to hear every juicy detail, and I wasn’t prepared for that. I didn’t show my best side there.	Allocating time for team dialog	Reflecting together on work practices	Team‐oriented strategies
… if I compare it to an emergency room, the adult emergency room, which I thought was tougher, it’s pretty nice here, we say to each other, “Yes, this went well, it worked out, and you did a good job and we worked well together”/…/if there’s a difficult incident/…/that we don’t yell at each other if something happens, but just say, “Maybe think about doing it this way,” or “it’s better, I have a suggestion,” so it doesn’t turn into mudslinging.	Providing feedback		
It was really fun to feel like, “Wow, how wonderful,” and also that we could have time to sit and laugh, and they could tell quite… well, both interesting and sometimes crude and sometimes funny or rowdy stories from working life, because, well, you need that outlet.	Informal debriefings		
But then I called a colleague who was below me, in the ward below, and asked, “How should I think about this?” And she was that supportive person who’s there for you/…/And it was one of those events that made me dare to ask questions, and why I’ll always help out when someone else comes and asks me, because it made me feel safe.	Collective problem‐solving	Working together toward a common goal	
And I know that we understand each other after so many years, and sometimes it was chaotic on the ward, and they would just say, “Hey, go in and do your thing, we’ll get you if we need you.” Then I didn’t have to go out, because they knew I always did my part, and when they knew it was chaotic out there, then it was chaotic for me in there too.	Identify ways of helping each other		
My boss, at least, she brought us in… together, and we got to sit down and talk about it, and, um… no, we didn’t really agree, I felt that she didn’t understand me regarding this. And then she went and talked to all her doctor colleagues about it/…/And I went to my colleagues, of course, and told them.	Handling conflicts between colleagues		

**TABLE 5:** Summary of main areas, categories, and subcategories.

Main areas (*n* = actions)	Categories (*n* = actions)	Subcategories (*n* = actions)
INDIVIDUAL‐ORIENTED STRATEGIES (*n* = 88)	Self‐managing the work situation (*n* = 50)	Adjusting work hours for personal reasons (*n* = 23)
		Changing jobs (=24)
		Receiving support from counseling professionals (*n* = 3)
	Sharing responsibility of the work situation (*n* = 38)	Communicating about personal matters with the immediate superior (*n* = 27)
		Customizing the working conditions (*n* = 11)

PROFESSIONALISM‐ORIENTED STRATEGIES (*n* = 57)	Adhering to a patient‐first approach (*n* = 36)	Prioritizing patients’ care needs (*n* = 13)
		Tailoring care to patients’ needs and preferences (*n* = 23)
	Continuing professional development in nursing (*n* = 17)	Taking career growth opportunities (*n* = 13)
		Creating a set of procedures for a successful nursing practice (*n* = 4)

TEAM‐ORIENTED STRATEGIES (*n* = 51)	Reflecting together on work practices (*n* = 17)	Allocating time for team dialog (*n* = 6)
		Providing feedback (*n* = 6)
		Informal debriefings (*n* = 5)
	Working together toward a common goal (*n* = 34)	Collective problem‐solving (*n* = 16)
		Identify ways of helping each other (*n* = 12)
		Handling conflicts between colleagues (*n* = 6)

## 3. Individual‐Oriented Strategies

The individual‐related strategies that nurses described concerned self‐managing their work situation and sharing responsibility for the situation with their superiors. Self‐management strategies included adjusting work hours for personal reasons, receiving support from counseling professionals, and changing jobs if needed. The rationales underlying these self‐management strategies were to safeguard nurses’ health and well‐being when their present nursing roles become unmanageable, even if this might curtail their career advancement. Shared responsibility for the work situation involved communicating personal matters with an immediate superior and customizing working conditions. A mutual approach led to enhanced engagement and sustainability in nursing, while a lack of mutual agreement resulted in distress.

### 3.1. Self‐Managing the Work Situation

#### 3.1.1. Adjusting Work Hours for Personal Reasons

Nurses described situations where tensions between work schedules, workplace routines, health needs, and family responsibilities required them to actively adjust their working hours to sustain both work participation and family life. They described two primary strategies for adjusting work hours for personal reasons. One was to work fewer hours, and the other was to work more hours. Working fewer hours was described as rooted in a need to meet family responsibilities, a desire to be a present parent in their children’s daily lives, or for health and recovery reasons. The latter typically involved changing their schedule to work fewer days a week or fewer night shifts, which nurses experienced as beneficial for recovery and a way of coping with deteriorating health when taking paid sick leave was not an option. Nurses also described working fewer hours as a strategy to fulfill work duties, for example, by working overtime, as such requests were experienced as part of the job and difficult to refuse.

Working more hours was experienced as a strategy to meet both work and family responsibilities for nurses using their parental leave rights (25% unpaid parental leave until the child turns 13), because working fewer hours per day was experienced as incompatible with work routines, such as debriefing at the beginning or end of a shift. Situations with a mismatch between scheduled working hours and work routines were described as ongoing problems that were almost always solved by working more hours than scheduled. These situations often ended with calls to their partners or children’s preschool about being late again and feelings of being a bad parent. This was considered detrimental to their well‐being, as well as to the health and well‐being of family members. Other examples included alternating between working more hours 1 week and fewer hours the next, to fulfill family responsibilities as a single parent. This was described as necessary for a sustainable nursing career, as it is otherwise difficult to combine nursing with single parenting. While these adjustments allowed nurses to maintain their participation in the profession, they often resulted in increased strain and tensions between work, health, and family life, thereby influencing the long‐term sustainability of their working lives.

#### 3.1.2. Changing Jobs

Nurses described situations in which persistent work pressures, limited control, and difficulty balancing work and family life made their current positions feel unsustainable. Changing jobs was a strategy to maintain or preserve their health and well‐being when their current job felt distressing or impossible to combine with family life. They described distress as arising from several sources, such as feeling a lack of control or cognitive overload because of the constant pressures of the job. The latter included having too much work to do with too few resources and being involved in conflicts with colleagues. In such situations, applying for a new job felt like the only option besides taking sick leave, which in some cases preceded the decision. In combination with work pressure, nurses also described how employers prioritized the organization’s operations over their well‐being as employees. For example, nurses would be asked to work endless overtime, even when managers saw they were exhausted. Starting a new nursing job, mostly working day shifts from Monday to Friday in community or primary care, was described as a boost for nurses’ health and work–family balance. Changing jobs was therefore a strategy that improved their health and work–family balance, thereby enabling continued participation in nursing. Nurses described, however, that changing jobs also involved trade‐offs, including fewer career opportunities and less stimulating work tasks, which could affect their long‐term workplace sustainability. Nevertheless, during the most demanding family years, these trade‐offs were considered acceptable.

#### 3.1.3. Receiving Support From Counseling Professionals

Nurses described situations in which the demands of their work caused emotional distress, while the working conditions themselves were perceived as difficult or impossible to change. In such situations, nurses described seeking private counseling as a self‐management strategy to help them manage their personal feelings and reactions to work, as the work itself felt difficult or impossible to change. Such support gave nurses strategies for dealing with the situation at hand and helped them manage their distress. Seeking private counseling provided nurses with strategies to manage their emotional responses, resulting in reduced distress and enabling them to continue working despite challenging conditions.

### 3.2. Sharing Responsibility for the Work Situation

#### 3.2.1. Communicating About Personal Matters With One’s Immediate Superior

Nurses described situations in which personal matters prompted them to communicate with their immediate supervisors. Personal matters that nurses communicated to their immediate supervisors primarily concerned health and family concerns, but also included other important issues, such as taking a leave of absence to fulfill personal dreams and aspirations. In some situations, communicating about health issues led to the initiation of a range of support or rehabilitation measures, such as assistance from the company’s healthcare system and adjustments to the work situation. Such a supportive response made it possible for nurses to remain in the workplace and fostered feelings of loyalty to the employer. In other cases—mostly related to family issues or conflicts at work, but sometimes also to health issues—the nurses felt that no action was taken because of a lack of mutual agreement on solutions. This was because supervisors did not meet their requests or did not provide the support they wanted. The same was described regarding nurses’ communication about feeling overwhelmed. These latter situations led to distress, reduced commitment to the organization, and decisions to resign.

#### 3.2.2. Customizing the Working Conditions

Nurses described situations in which their health needs or desire for greater flexibility and stimulation required adjustments to their working conditions. For those with health problems, a customized work situation could be either a rehabilitation strategy for returning to work after a spell of sick leave or a habilitation strategy for remaining in the workforce with optimal functionality. For nurses needing a more flexible or stimulating work situation, strategies commonly involved combining two different positions or tasks, such as working part‐time in two different types of care, or combining administrative and bedside tasks. The latter included working remotely one or 2 days a week. This allowed nurses to sustain their enjoyment of nursing and to have a more manageable work situation, both of which had a positive impact on their sustainability in the profession.

## 4. Professionalism‐Oriented Strategies

The professionalism‐oriented strategies that nurses described involved adhering to a patient‐first approach and having ongoing professional development in nursing. Adhering to a patient‐first approach meant prioritizing patients’ care needs, which gave nurses a sense of professional pride, despite the ethical stress caused by organizational constraints. It also meant tailoring care to patients’ needs and preferences, and such a person‐centered care approach enhanced nurse satisfaction. Ongoing professional development in nursing entailed taking opportunities for career growth and creating a set of procedures for successful nursing practice. These strategies provided security and control in crucial situations, supporting excellence and motivation to remain in nursing.

### 4.1. Adhering to a Patient‐First Approach

#### 4.1.1. Prioritizing Patients’ Care Needs

Nurses described situations in which high workload and organizational constraints challenged their ability to provide the level of care they considered necessary for patients. Their descriptions of such situations included actions taken in extraordinary situations, such as trauma, but also in long‐term rehabilitation care. In these situations, nurses described strategies for always putting patients’ care needs first, despite other organizational problems, such as too many patients being admitted. These actions evoked both job satisfaction and pride in doing what matters most to patients. Job satisfaction was, in turn, described as vital to long‐term working life sustainability. Nurses also described situations in which they were overwhelmed by work pressure but continued working to the best of their ability in order to prioritize the patients’ needs. These situations led to distress and made them question their ability to remain in nursing. The urge to always prioritize patients’ care needs created ethical stress when organizational factors did not allow nurses to act in accordance with this priority. When ethical stress became a daily experience, it also led to doubts about the sustainability of a nursing career or became a reason for resignation.

#### 4.1.2. Tailoring Care to Patients’ Needs and Preferences

Nurses described situations in which they sought to tailor care to patients’ individual needs and preferences, often within organizational or medical constraints. Providing tailored care involved establishing a working alliance with patients and listening to their personal wishes and preferences. This approach also involved supporting their significant others, such as when they were in hospice or pediatric care. Engaging with patients in both short‐ and long‐term care was described as a source of joy in nursing. Examples of tailored care include situations where nurses could act in accordance with the patients’ wishes, which provided job satisfaction, as well as situations where this was not possible, due to conflicting medical orders. The latter type of situation caused ethical distress. For example, during the COVID‐19 pandemic, a patient did not want to be placed on a ventilator, but the nurse was forced to act against the patient’s will because of the physician’s orders.

Tailoring care to patients’ needs also meant providing them and their significant others with timely and relevant information. Such information was psychoeducational and aimed at increasing the patients’ health literacy in relation to a diagnosis or illness, or informing them about the course of treatment. Information was delivered with respect to patients’ needs and preferences, and if it was acknowledged as such by the patients, it provided reassurance that a good job was being done. Nurses’ descriptions of tailored care also included follow‐up support, for example, meeting with patients and significant others after traumatic or acute care situations and listening to their experiences of the care they received. When the patients described feeling safe and satisfied with the care provided, nurses felt proud to have provided excellent nursing. When such care could be provided, nurses experienced pride and job satisfaction, which they viewed as contributing to a sustainable nursing practice. Conversely, barriers to acting according to patients’ wishes resulted in ethical distress and challenged sustainability in nursing.

### 4.2. Continuing Professional Development in Nursing

#### 4.2.1. Taking Opportunities for Career Growth

Nurses described situations in which opportunities for career development arose, either from desires or organizational needs that they identified and communicated to their superiors, or from suggestions made by superiors to address organizational needs. The latter included being asked to undertake specialist training or to take on responsibilities at an organizational level, for example. This could mean being in charge of technical equipment, procedures, or methods used in nursing, or providing training to colleagues. Receiving a positive response to a request from supervisors or being selected for more responsibility was gratifying. They also saw it as recognition of being an excellent nurse, which supported their decision to stay in nursing. This was especially the case when, after completion of education or training, the new role and/or responsibilities were designed or adapted to match their personal strengths and qualities. Such personalized roles or organizational assignments helped them to excel and be successful in nursing. When opportunities aligned with nurses’ strengths and interests, they fostered recognition, professional growth, and commitment to remain in nursing. But nurses also described how, in some cases, their original duties had expanded over time, leading to a loss of control and less time to spend with patients and on core nursing tasks. This eventually led to distress, questioning of the sustainability of the work situation, and doubts about remaining in the present role.

#### 4.2.2. Creating a Set of Procedures for a Successful Nursing Practice

Nurses described situations in which recurring challenges in clinical practice required them to develop structured ways of responding to complex or demanding care situations. This included creating a set of procedures that they used to provide excellent care. Examples include prepared responses to be used in telephone counseling with patients who questioned the advice provided by the guidelines, because being challenged several times a day felt devastating to nurses’ well‐being. It also included codifying the skills and experience that nurses had acquired over time for use in both ordinary and extraordinary care situations. For the latter, having a set of procedures to follow was seen as essential, as everyone needed to know what to do in such situations. It also needed to be synchronized, as the patient’s life depended on it. Having such procedures and being able to use them to facilitate the work provided a sense of security and control, of knowing what you were doing and why, thereby supporting nurses’ ability to sustain their motivation to work in demanding clinical situations. This, in turn, created a sense of excellence in nursing and a desire to remain in nursing.

## 5. Team‐Oriented Strategies

The team‐oriented strategies that nurses described involved reflecting on work practices together and working toward a common goal. Reflecting together on work practices was facilitated by allocating time for team dialog, feedback, and informal debriefings. These activities were considered vital for fostering a positive team culture, promoting nurses’ engagement and joy in their work, and facilitating their professional growth. Working together toward a common goal entailed engaging in collective problem‐solving, which improved patient care and job satisfaction, as well as identifying ways of helping each other, which fostered teamwork and a sense of control. It also involved handling conflicts between colleagues, because conflicts negatively affected teamwork and job satisfaction, and by extension, workplace sustainability.

### 5.1. Reflecting Together on Work Practices

#### 5.1.1. Allocating Time for Team Dialog

Nurses described situations of reflecting together on work practices through team dialog, which was needed to manage everyday challenges and process demanding clinical events. Allocating time for team dialog was described as important for both the day‐to‐day work and for deciding on a course of action for future work. For example, regular workplace meetings were considered beneficial for agreeing on workplace rules, and the dialogs they facilitated were considered essential for fostering a positive working climate and doing good patient work. Team dialog was also seen as necessary after extraordinary situations in order to debrief and reflect on what had happened. Otherwise, nurses might not be able to let go of it, which could negatively impact their work or family life. Nurses described several situations in which team dialog helped them process their experiences and feelings. However, they also described occasions when the team dialog felt excessive, because it was superficial or not relevant to the situation at hand. Team dialog most often provided intra‐ and interprofessional support, but situations were also described in which the different professions involved in the teamwork questioned each other. Such questioning led to feelings of anger and resentment, which was in sharp contrast to the supportive dialogs. The type of dialog was therefore crucial; meaningful dialog fostered support, learning, and a positive work climate that helped nurses sustain their work, whereas superficial or conflictual discussions undermined sustainable working life.

#### 5.1.2. Providing Feedback

Nurses described situations in which giving and receiving feedback within the team was important for learning, professional development, and maintaining a supportive team culture. They described providing feedback to junior colleagues to help them learn and excel in their nursing practice, and how this made them feel validated and appreciated for their nursing skills. Nurses also described receiving feedback from other team members and how they navigated these interactions to ensure that the feedback was communicated constructively and supported learning rather than undermining confidence. Feeling belittled by feedback provided in a scolding or ranting tone, for example, led to insecurity and reduced job satisfaction. This was described as especially true when they were new to the job or on days when their energy levels were already low. Constructive feedback provided both learning opportunities and job security, which made nursing more sustainable for them in the long run, whereas belittling or harsh feedback reduced their motivation to remain in the profession.

#### 5.1.3. Informal Debriefings

Nurses described situations in which informal debriefings with colleagues were used to reflect on their everyday practice. These informal debriefings provided them with learning opportunities and a sense of joy in nursing. Such debriefings took place after memorable situations, when it was necessary to reflect on what had happened in order to learn from it or be reassured that they had done the right thing. They also took place in the day‐to‐day work when there was a need to debrief about patients, colleagues, or the organization. Being able to reflect together with team members or colleagues offered a sense of relief when the work felt difficult. It was comforting for nurses to have colleagues with whom they could laugh and talk about past mistakes. In such situations, they also learned from each other’s mistakes and coached each other. Examples of informal debriefing also included sharing the joy of providing excellent care together. These informal reflections fostered collegial support and shared learning that helped nurses manage difficult experiences and sustain their engagement in nursing.

### 5.2. Working Together Toward a Common Goal

#### 5.2.1. Collective Problem‐Solving

Nurses described various situations in which complex clinical challenges required them to engage in collective problem‐solving with their colleagues. These situations encompassed both intraprofessional and interprofessional collaborative efforts in nonacute and acute situations. Examples include collectively ascertaining the underlying issue afflicting a critically ill child or performing CPR. Collective problem‐solving involved asking each other for advice and support in situations where nurses felt uncertain or lacked experience, as well as performing bedside care together. Interprofessional collective problem‐solving was facilitated by skilled and experienced colleagues, face‐to‐face meetings instead of digital communication, and a work climate in which the shared goal was to do what was best for the patient. In such a climate, nurses felt comfortable asking for support and were confident that they would receive it, regardless of whether they asked a nurse, a physician, or a nurse assistant. Such collaborative problem‐solving fostered confidence, mutual support, and effective patient care, enabling nurses to manage demanding situations and continue working in nursing.

#### 5.2.2. Identifying Ways of Helping Each Other

Another aspect of working toward a common goal was identifying ways to help each other. Nurses described situations in which this was necessary in order to manage workload and ensure safe patient care. It involved planning the work together with colleagues at the beginning of the workday to identify work situations in which they need help, either due to the patient’s support needs or their own uncertainty about their skills. It also involved asking colleagues for support in the moment or offering to help others without being asked. An example of the latter would be for nursing assistants to prepare nurses’ tasks to support them in stressful situations. These situations were characterized by feelings of relief and a sense of greater control. Nurses also described situations in which they helped other colleagues, citing examples such as backing up colleagues at the bedside to allow them to use the restroom, and preventing a colleague from making a fatal mistake. Being able to help each other was satisfying and fostered a sense of teamwork. Mutual support provided relief and strengthened teamwork, supporting nurses’ ability to sustain their work in demanding care situations.

#### 5.2.3. Handling Conflicts Between Colleagues

Nurses described situations in which conflicts with colleagues created tensions in everyday collaboration. Such conflicts were seen as a downside of teamwork that impacted their sense of well‐being and job satisfaction. These conflicts could be between nurses or between nurses and physicians. Examples of the latter involved arguments about the most effective ways to coach patients in providing self‐care. Avoiding working together was a result of these conflicts, and if it was not possible to avoid colleagues, nurses pretended that they were not there. Nurses described such avoidant behavior as energy‐depleting. They also described conflicts as spreading from those originally involved to the entire team, which caused a negative atmosphere. Sometimes, conflicts were handled in tripartite dialogs with superiors, but these measures were not effective, leading nurses to resign or consider resigning to avoid the conflict. Such unresolved conflicts depleted nurses’ energy, weakened the team climate, and in some cases led nurses to consider leaving their positions, thereby undermining sustainable working life in nursing.

## 6. Discussion

The findings suggest that nurses sustain their working lives through the interaction between individual‐, professionalism‐, and team‐oriented strategies for sustainability, but that these strategies are highly dependent on organizational conditions. Each of them encompasses specific actions with implications at the individual and organizational levels. Overall, these strategies highlight the importance of striking a balance between individual needs, professional growth, and team dynamics to promote sustainable nursing careers amidst organizational challenges.

### 6.1. Individual Level

At the individual level, the individual‐ and professionalism‐oriented strategies that nurses employed had consequences for their well‐being, professional commitment, and willingness to remain in nursing. Previous studies have shown that achieving work–life balance is a significant challenge for nurses, which can lead to nurse turnover [30, 34]. It is particularly significant for nurses working under certain conditions, such as in intensive care units [35]. The findings of the current study add nuance to this literature by showing that nurses may also actively self‐manage their work–life balance and make adjustments in order to remain in nursing. The self‐management strategies used by nurses to promote work–life sustainability, such as adjusting their working hours, seeking support from counseling professionals, or changing jobs, could be considered self‐care strategies. Self‐care, defined as a “multidimensional, multifaceted process of purposeful engagement in strategies that promote healthy functioning and enhance overall well‐being” ([36], p. 326), is increasingly recognized as crucial for preventing and managing workplace stress and burnout among nurses [37]. In the current study, such strategies helped mitigate stress and promote recovery, thereby supporting nurses’ overall well‐being and sustainability in nursing. From this perspective, the strategies described by nurses can be understood as active efforts to protect their health and sustain their engagement in nursing over time.

At the same time, the findings raise questions about the extent to which sustainability in nursing is currently maintained through individual adaptation rather than through supportive organizational conditions. Strategies such as changing jobs or privately seeking counseling suggest that nurses may compensate for insufficient organizational support through personal coping efforts. In this sense, self‐care may function as both a resource and an indicator of unsustainable working conditions.

The finding that nurses changed jobs to self‐manage contradicts Leineweber et al.’s [9] finding that the main reasons for leaving the nursing profession are factors at the individual level. Notably, changing jobs was not primarily described in terms of leaving the profession, but as a strategy for remaining within it, suggesting that mobility within nursing may represent an important mechanism for sustaining careers when specific work environments become untenable.

A need to self‐manage the work situation was also related to cognitive overload, as both new and experienced nurses described. Collins [38] has emphasized the importance of nurse leaders recognizing cognitive overload, because it impacts nurses’ well‐being, which can lead to errors that impact patient safety and quality of care. The cognitive overload described in the current study was linked to job pressures and conflicts with colleagues, suggesting that the issue is not only cognitive, but also relational and organizational. The role of workplace conflict further illustrates how relational work environments influence sustainability in nursing. Previous research has demonstrated that exposure to workplace conflict is associated with increased risk of mental distress [39], as well as an increased risk of taking sick leave [40]. The current findings extend this knowledge by showing how unresolved conflicts, particularly when combined with inadequate support from supervisors, may erode nurses’ professional commitment and ultimately lead to an intention to leave the workplace.

These findings indicate that leadership plays a critical role in shaping sustainable working conditions. Rather than relying on nurses’ individual coping strategies, proactive leadership should address the underlying organizational conditions that generate work–life imbalance, cognitive overload, and interpersonal conflicts. Support should also recognize sustainability challenges throughout the course of a career, including periods of cognitive overload experienced when entering a profession or when nearing retirement, and work–life conflicts, for example, during child‐rearing years.

Professionalism‐oriented strategies with individual‐level outcomes included practicing a patient‐first approach and taking advantage of opportunities for professional development in nursing. In the current study, providing person‐centered care was closely linked to nurses’ sense of pride and satisfaction in their work, which aligns with previous research demonstrating a positive correlation with nurses’ job satisfaction [41]. However, the findings also suggest that the relationship between person‐centered care and a sustainable working life is more complex than a simple correlation with job satisfaction. Nurses described a person‐centered approach and tailoring care to patients’ needs and preferences as central to their professional identity. The opportunity to practice in accordance with professional values thus appears to function as an important source of meaning and motivation in nursing. This interpretation aligns with the person‐centered care framework, where professional competence is considered a key prerequisite for person‐centered practice [42], and has been shown to be positively related to person‐centered care [43]. The current findings extend this perspective by illustrating how opportunities to practice person‐centered care may support nurses’ long‐term engagement in the profession. However, the findings also indicate that such practices depend on organizational conditions, including access to time, resources, and support.

This could be linked to the importance of structural empowerment—defined as access to empowering conditions such as information, resources, support, and opportunities [44]. When such empowering conditions were present, nurses experienced a sense of purpose and professional pride, which in turn influenced their long‐term sustainability in nursing.

Professionalism‐oriented strategies involved taking advantage of opportunities for career development, such as specialization training and taking on organizational responsibilities, and contributed to nurses’ personal growth and excellence in nursing. In the current study, these opportunities were often described as receiving recognition from supervisors and colleagues, which further boosted nurses’ commitment to their profession. Hence, recognition appeared to play a particularly important role, suggesting that professional development is not only related to competence‐building but also to professional identity. This interpretation aligns with studies, indicating that ongoing professional development enhances nurses’ motivation and dedication, as well as job satisfaction [45, 46].

However, the findings of the current study also indicate that the relationship between career development and sustainable working life is not straightforward, and opportunities for professional development and career progression can become a source of strain if they are not aligned with individual capacity and workload. Other studies have found that training and education emerge as a cross‐cutting theme throughout nurses’ careers [46], but the goals, motivations, and needs that nurses must have in order to direct and participate in their continuing professional development may vary according to their age and position [47]. This was also evident in the current study, which highlights the importance of providing individualized support during career transitions and helping nurses who have undergone specialist training find organizational assignments that build on their personal strengths and qualities. Consequently, providing individualized career paths and aligning specialist competencies with appropriate organizational roles may be critical for supporting long‐term engagement and preventing professional stagnation or overload, thereby promoting sustainable nursing careers. In this sense, career development can be understood not merely as professional advancement, but as an organizational strategy for sustaining nursing careers over time.

Overall, the above‐mentioned individual‐ and professionalism‐oriented strategies and their consequences influence the sustainability of nurses’ working lives at an individual level, affecting their health and well‐being, professional identity, engagement, and commitment to nursing. However, the findings also suggest that the effectiveness of such strategies cannot be understood solely at the individual level. As previous studies have shown, self‐care strategies are influenced by a complex interplay of processes beyond the control of individual nurses. For example, the work environment, collegiality, team dynamics, and professional identity of nurses have been illuminated [48]. The current study reinforces this perspective by demonstrating that the consequences of nurses’ strategies are highly contingent on organizational conditions. Whether a strategy becomes beneficial or detrimental for sustainability largely depends on the organizational environment, the availability of support, and nurses’ opportunities to effectively employ these strategies in the workplace. This suggests that sustainability in nursing cannot be achieved through individual effort alone. Rather, nurses’ strategies interact with structural and relational conditions that either enable or constrain their capacity to maintain well‐being and professional engagement over time. From this perspective, sustainable working life emerges as not simply a result of individual coping, but as a dynamic process shaped by the interplay between individual agency and organizational context.

### 6.2. Organizational Level

At the organizational level, the findings highlight the central role of organizational support in shaping nurses’ ability to sustain their working lives. Supportive leadership and a positive organizational culture influenced whether the strategies nurses employed resulted in beneficial or detrimental outcomes for their sustainability in nursing. This observation reinforces previous research identifying the importance of organizational support for retaining nurses (e.g., [11]), with supportive leadership in particular having been shown to positively affect work‐related well‐being [49] and the retention of nurses [50]. In particular, leadership characterized by a “person‐first” approach, in which leaders acknowledge personal circumstances and customize working conditions to match individual needs, appeared to support job satisfaction, commitment, and retention. From a sustainability perspective, this suggests that leadership practices function as enabling conditions for aligning professional demands with personal needs across the career span.

The understanding of nurses’ support needs for a sustainable working life can be expanded by reflecting on supportive leadership in relation to social support theory [51], i.e., in terms of instrumental, informational, emotional, and belonging support. Applying this framework helps illuminate how supportive leadership may function as a key mechanism through which sustainable working lives in nursing are enabled. In the current study, supportive leadership was not only described in terms of managerial actions, but also in terms of nurses and leaders taking shared responsibility for the nurses’ work situation and ongoing professional development over time.

Instrumental support involved providing practical support, such as adjusting the work situation to make it more manageable. This was particularly important when nurses experienced health challenges or changing life circumstances, indicating that flexible work roles may be essential for sustaining participation in nursing across the life course. Informational support involved helping nurses navigate stressful or challenging situations, such as team conflicts, highlighting how leadership can help nurses navigate organizational pressures that might otherwise undermine their well‐being. This kind of support is also needed throughout a nursing career, as such situations can occur at any time. Emotional support involved encouraging nurses by recognizing their personal strengths, qualities, career plans, and preferences, to make them feel valued. This was important throughout working life, especially after gaining experience or making efforts to develop professionally.

Belonging support was important at all times throughout a nursing career. Nurses emphasized the importance of leaders adopting a person‐first approach (i.e., placing the individual ahead of the organization) and of being recognized as an integral member of the organization rather than merely a resource within it. Such recognition fostered a stronger sense of involvement and commitment. Taken together, these findings underscore the pivotal role of leadership practices in shaping organizational conditions that sustain nurses’ long‐term engagement in the profession.

Organizational support for high‐performance teamwork also emerged as a key condition for sustainability. Implementing team‐oriented strategies such as collective problem‐solving effectively improved teamwork, promoted a positive work climate, and influenced job satisfaction, commitment, and nursing practice. In this way, teamwork functioned as a relational resource that enabled nurses to sustain their engagement in demanding work environments. These findings are consistent with previous research demonstrating the importance of effective teamwork, in which team members experience psychological safety [52]. The importance of psychological safety was also emphasized by the nurses in the current study, suggesting that psychologically safe team environments may be particularly important in professions characterized by high responsibility and complex decision‐making. At the same time, the findings illustrate that teamwork is shaped by broader organizational and relational dynamics. Efficient teamwork is influenced by power structures, for example, which can create barriers to interprofessional teamwork [53]. This tension was also visible in the current study, in which conflicts between physicians and nurses were described as leading to energy‐depleting avoidance behavior. These findings therefore suggest that, while teamwork can function as an important sustainability resource, its effectiveness depends on organizational conditions that support respectful collaboration and address hierarchical barriers within healthcare teams.

The findings also highlight the importance of organizational culture in shaping nurses’ ability to sustain their working lives. Organizational culture, defined as “the way things are done within hospital organizations” ([54], p. 3), may influence how nurses interpret and respond to work demands. Previous research has linked organizational culture to work‐related stress, particularly when leadership practices and management approaches are perceived negatively, contributing to such issues as sick leave, overtime, and occupational injuries [54]. The current study adds nuance to this understanding by illustrating how cultural norms around organizational priorities within healthcare organizations may either reinforce or challenge nurses’ capacity to remain in the profession.

In situations where organizational priorities appeared to outweigh nurses’ well‐being—such as when supervisors requested overtime despite visible exhaustion—nurses interpreted this as an organizational culture that puts the organization first, rather than the nurses. Such experiences were described as having detrimental consequences for nurses’ health and well‐being, suggesting that organizational cultures that solely emphasize productivity or operational continuity may inadvertently undermine long‐term workforce sustainability.

In contrast, a culture emphasizing professionalism and patient‐centered practices was highlighted as beneficial for sustainability, indicating that organizational cultures aligned with professional values may function as important motivational resources. This finding is consistent with previous studies, demonstrating that nurses are willing to stay when the workplace culture and working conditions align with their personal and professional needs [11]. From a sustainability perspective, this suggests that organizational culture not only shapes immediate work experiences [55], but also influences nurses’ long‐term engagement and willingness to remain in the profession.

Overall, the findings suggest that the organizational‐level consequences of nurses’ sustainability strategies are multifaceted and closely tied to the conditions in which these strategies are enacted. When organizational environments support nurses’ efforts—through responsive leadership, flexible work arrangements, and recognition of individual needs—these strategies appear to strengthen professional commitment and facilitate retention. In this sense, nurses’ strategies can function as resources that enable organizations to maintain a stable workforce. However, the findings also indicate that when such efforts and needs are unsupported or ignored, the consequences may be detrimental not only for nurses, but also for the organization. As seen in the findings in the current study, organizational neglect leads to nurses planning to leave and ultimately to higher turnover. From a sustainability perspective, this suggests that organizations cannot rely solely on nurses’ individual efforts to maintain their working lives. Rather, leadership practices and organizational policies need to actively support these strategies by fostering person‐centered leadership, recognizing nurses’ evolving needs across the career span, and creating conditions that allow nurses to sustain both their professional engagement and their well‐being over time. Therefore, organizations should foster supportive leadership and implement “person‐first” policies that reinforce these strategies to ensure sustainable nursing careers and retention.

### 6.3. Strengths and Limitations

The most important strength of this study is its in‐depth focus on nurses’ strategies for a sustainable working life.

To enhance credibility, the participants were informed that the study focused on a healthy and sustainable working life rather than on risk factors. This may have caused the participants to overemphasize the positive aspects of their work life in their responses. However, during data collection, it was observed that participants tended to notice negative, sustainability‐related events more than positive ones. The tendency to overemphasize adverse experiences led to situations where both positive and negative consequences were included in the analysis, providing a comprehensive understanding of nurses’ working life sustainability and job retention.

The interviews were conducted by an experienced interviewer with expertise in behavioral science, but not nursing. This outsider perspective was considered both a limitation and a strength. While it limited insider insights, it also reduced the risk of important data being left unspoken, due to shared professional backgrounds and experiences. The interviewer’s approach included detailed follow‐up questions and resulted in rich, in‐depth descriptions of the participants’ experiences. Summaries of the interviews were shared with the participants for member checking, further strengthening the credibility of the study.

Regarding dependability, the study employed the CIT method, which involves systematic data collection, analysis, and reporting [28, 29]. This approach recommends collecting at least 100 critical incidents, with each participant reporting two or three incidents. In this study, participants described varying numbers of situations, ranging from one situation containing several events (e.g., being a new nurse with events concerning onboarding practices, leadership, and collegial support) to several different situations. The sample size was sufficient to meet the recommended number of significant situations for each participant, and the interview data were rich enough, with detailed descriptions of situations, actions, and outcomes, to ensure the quality of the data collection.

Dependability was further strengthened through a structured and transparent analytical process. The first author conducted the initial coding and identification of important situations, after which the emerging patterns, subcategories, and categories were continuously discussed within the research team to ensure consistency and coherence in the interpretation. This iterative process allowed the researchers to compare interpretations and refine the categorization until consensus was reached. In addition, the analytical decisions and category development were documented throughout the process, creating an audit trail that enhances the transparency and traceability of the analysis.

Confirmability was ensured by grounding the findings in the participants’ descriptions of important situations and actions. The analytical process followed the systematic procedures of the CIT, enabling clear links between incidents (situations), actions, and outcomes. The analysis was conducted by a collaborative team of researchers with diverse expertise in nursing and behavioral science. This collaborative approach allowed different perspectives to be considered during the analytical process and helped to mitigate individual biases. Through ongoing discussions and critical reflection within the team, interpretations were continuously examined and refined to ensure that the findings remained grounded in the empirical data, rather than in the researchers’ preconceptions. In addition, representative quotations are presented in the results to demonstrate how the findings are rooted in the empirical data.

The transferability of the findings was enhanced by selecting a broad sample of nurses to capture a range of situations in different healthcare settings related to sustainability in working life. However, while the sample included participants with varying skill levels and specialties, not all care areas were represented. Furthermore, the limited number of participants from specific contexts may restrict the depth of the analysis in relation to different healthcare settings. As the study primarily included experienced nurses, alongside a few newly qualified nurses, recall bias may have influenced the findings. Nevertheless, some participants recalled detailed experiences from the early stages of their careers, suggesting that the findings are robust across different lengths of time in the profession.

## 7. Conclusions and Implications

Nurses employed a combination of individual‐, organizational‐, and team‐oriented strategies to promote their health and well‐being and ensure a sustainable working life. Throughout their careers, the strategies and actions of nurses in situations important to sustainability in working life seemed to be evolved or adapted in response to different individual, organizational, and societal conditions. From this perspective, sustainability in nursing may be understood as a dynamic process that develops over time rather than as a fixed state. Adopting a long‐term sustainability perspective may therefore be useful when considering nurse retention, to ensure sustainable working lives.

These findings contribute to a more nuanced understanding of how nurses can maintain sustainable careers in healthcare and have implications for organizations and nurse leaders. First, the findings emphasize the importance of taking a multifaceted approach to supporting nurses’ strategies for enhancing sustainability, such as by utilizing personal strengths, attending to individual needs, providing customized professional development, and fostering supportive team dynamics. Second, the findings indicate that organizational support is pivotal in enabling such strategies. In particular, supportive leadership employing a “person‐first” approach and a positive organizational culture fostering work–life balance appears to create an environment conducive to nurse sustainability. Third, the study highlights how negative organizational cultures and work environments have detrimental consequences such as excessive overtime demands and neglect of the individual within the organization. Such conditions influence nurses’ health, well‐being, and willingness to remain with the organization. Consequently, organizational policies aimed at reducing organizational stressors, such as excessive workloads and ethical conflicts, are important for long‐term sustainability in nursing.

Taken together, these findings illustrate the multifaceted nature of sustainability in nursing and suggest a need for integrated strategies that address individual, professional, and team dynamics throughout a nursing career. A dynamic perspective on sustainability may therefore provide a useful lens for understanding how nurses navigate the conditions that shape their long‐term engagement in the profession.

## Funding

The study was funded by AFA Försäkring (AFA Insurance), Dnr 210233.

## Disclosure

The funder has no influence over the results and visualization of the data.

## Conflicts of Interest

The study is funded by AFA Försäkring (AFA Insurance).

## Data Availability

The data that support the findings of this study are available upon request from the corresponding author. The data are not publicly available due to privacy or ethical restrictions.
